# Effects of Dietary Protein-to-Energy Ratios on Growth, Immune Response, Antioxidative Capacity, Liver and Intestinal Histology, and Growth-Related Gene Expression in Hybrid Yellow Catfish (*Pelteobagrus fulvidraco ♀ × Pelteobagrus vachelli ♂*)

**DOI:** 10.1155/2023/9106332

**Published:** 2023-05-23

**Authors:** Sehrish Taj, Quan Han, Xiaoyi Wu, Haoran Yin, Lixia Tian, Huijun Yang, Yongjian Liu, Junwa Huang

**Affiliations:** ^1^State Key Laboratory of Marine Resource Utilization in South China Sea, Hainan University, Haikou 570228, China; ^2^Department of Aquaculture, Ocean College of Hainan University, Haikou 570228, China; ^3^Hainan Provincial Key Laboratory for Tropical Hydro biology and Biotechnology, Department of Aquaculture, Hainan University, Haikou, Hainan 570228, China; ^4^Guangzhou A Share Aquatic Science and Technology Co. LTD, China

## Abstract

This study is aimed at evaluating the effects of dietary protein-to-energy ratios on the growth, immunological response, antioxidative capacity, liver and intestinal histology, and growth-related gene expression of hybrid yellow catfish (*Pelteobagrus fulvidraco ♀ × Pelteobagrus vachelli ♂*). Eight diets were formulated to form different protein/energy ratios of 84, 88, 90, 93, 95, 96, 99, and 103 mg/kcal (P/E84, P/E88, P/E90, P/E93, P/E95, P/E96, P/E99, and P/E103), respectively. These diets contain different levels of gross energy (GE), ranging from 4.13 to 4.76 kcal g^−1^. Seven hundred and twenty healthy fish (17.15 ± 0.02 g) were randomly dispersed into 24 rectangular fiberglass tanks with 8 treatments in triplicate groups. The fish fed a P/E ratio of 95 mg/kcal demonstrated the best growth and feed utilization. A significant (*P* < 0.05) increase in percent weight gain (WG%) and specific growth rate (SGR) was seen as the dietary P/E ratio ameliorated from P/E84 to P/E95, followed by a decreased pattern in these parameters. Feed conversion ratio (FCR) and daily feed intake (DFI) were significantly impacted by dietary P/E ratios (*P* < 0.05). Additionally, an optimum P/E ratio improved intestinal morphology. However, low or high P/E ratio diets can cause oxidative stress, impaired liver function, and significantly reduced nonspecific immunity. The expression of target of rapamycin (TOR) and insulin-like growth factor-1 (IGF1) genes in the liver was considerably influenced by dietary protein-to-energy ratios (*P* < 0.05). Based on the statistical analysis of WG% against the dietary P/E ratio, the optimal P/E ratio for the studied species was estimated to be 92.92 mg/kcal.

## 1. Introduction

Considerable improvements in aquaculture production over the past two decades have enhanced the quality of the global population to consume a variety of nutritious food [1, 2]. Fish products are highly valued sources of animal protein, making up between 50 and 60 percent of the daily protein intake for humans [3]. However, as the population grows and more people become aware of their health, there is an increasing demand for fish and other items associated with fishing [4]. However, in aquaculture, feed ingredients are the most expensive when it comes to protein [5, 6]. Therefore, it is imperative to establish data on the nutrient requirements of fish, as this could be helpful in formulating affordable feeds and generating profitability through aquaculture [7–9].

One of the significant elements in the composition of high-quality feed is the protein-to-energy (P/E) ratio [10]. A common way to express this P/E ratio is as digestible protein over digestible energy (DP:DE). For popular aquaculture species, the ideal DP:DE ratio lies between 18 and 23 g MJ^−1^ (43 and 55 mg/kcal) [11, 12]. Since protein and energy digestibility in most formulated diets are comparable, the ideal range of DP:DE is similar to the P/E [13]. It has been demonstrated in other fish species that sufficient dietary nonprotein energy can spare dietary protein [14, 15]. It is obvious that when the P/E ratio of the diet is unbalanced together with insufficient nonprotein energy, fish may utilize dietary protein as an extraenergy source in order to ensure maintenance prior to growth [16], which results in lower growth and productivity [17]. A surplus of nonprotein energy, however, might reduce feed intake [18, 19], results in fatty fish or liver fat deposition [20], and also prevent the ingestion of other nutrients [21]. Therefore, in order to ensure optimal protein deposition, aquaculture feed must contain a balanced protein-to-energy ratio [22].

Nutritional deficiencies can impair immunity, oxidative status, and animal performance if fish species are not provided with sufficient macronutrients in their diet [23, 24], as well as cause several pathological changes in the morphology of liver and intestine tissues [25, 26]. Proper protein-to-energy ratios in diets have the potential to negatively impact intestinal morphology, which affects nutrient absorption and digestion, as well as liver function and morphology. These effects may depress the immune system and raise the risk of infectious diseases [26–29]. Lipid is deposited in vacuoles, which results in steatosis primarily in the liver, but it can also build up in enterocytes, as dietary lipid levels surpass the oxidation capability. There is evidence to support these effects on a wide variety of fish species, including juvenile shi drum (*Umbrina cirrosa*) and meagre (*Argyrosomus regius*) [29, 30]. Hence, finding an ideal dietary protein-to-energy ratio is so essential to a fish's health and physiological condition.

Studies of fish protein metabolism with molecular tools have also revealed new and more precise information. Protein synthesis and deposition are significant components in the determination of the growth and development of aquatic animals [31, 32]. One of the important genes is IGF-1 which modulates cell growth and proliferation by triggering the TOR signaling pathway [33]. Since translation initiation is the first step in the TOR pathway, it is crucial for controlling protein synthesis [34]. By means of the TOR pathway, IGF-1 regulates protein production and destruction [35]. Although TOR signaling is complex and poorly understood in fish, several studies have shown that it could be affected by dietary protein levels, just as they do in mammals [36, 37]. Therefore, it is imperative to understand if decreases in nutrient efficiency are related to changes in the transcription of genes involved in protein metabolism because the liver is involved in nutrient metabolism.

The hybrid yellow catfish, whose origin stems from cross-breeding between the third generation of female *P. fulvidraco* and the second generation of male *P. vachelli*, have high artificial fertility rates, high frequency of emergence, improved growth performance, and hypoxia tolerances than the original fish [38], and they are becoming increasingly popular in China and Southeast Asia [39]. Hence, hybrid yellow catfish have proven to be well suitable for commercial purposes, leading to an increase in hybrid production. Global production of *Peltobagrus fulvidraco* reached 417,347 tons in 2016 and continues to increase [40]. Currently, research on hybrid yellow catfish is primarily focused on reproductive biology and artificial breeding [41, 42], genetics [43], and biochemical composition [39, 44, 45].

A knowledge gap exists due to a lack of research on the optimal protein-to-energy ratio (P/E ratio) of hybrid yellow catfish. It is therefore imperative to develop diets that contain appropriate amounts of P/E ratio to maximize the growth of this emerging aquaculture species. Such an establishment can progressively help aquaculture sustainability in the face of increasing demand on local, regional, and global levels. Thus, this study provides a thorough understanding of the fundamentals and a reference for formulating a unique diet for hybrid yellow catfish with balanced nutrition.

## 2. Materials and Methods

### 2.1. Ethics Approval

Experimental manipulations and the methodological protocol were approved by the expert ethics committee. The Guide for the Care and Use of Laboratory Animals in China was followed when taking samples.

### 2.2. Diet Preparation

Eight diets were formulated to contain different protein/energy ratios of 84, 88, 90, 93, 95, 96, 99, and 103 mg/kcal (P/E84, P/E88, P/E90, P/E93, P/E95, P/E96, P/E99, and P/E103), respectively. These diets produce different levels of gross energy, ranging from 4.13 to 4.76 kcal g^−1^. To complete the formula, carbohydrates were added, with low-protein diets containing more carbohydrates (>20%) than high-protein diets (<20%). The diet formulations along with proximate composition are presented in Table 1. Firstly, all dry ingredients were weighed and mixed for 30 minutes in a Hobart mixer. Lipids were gradually added during constant mixing. After that, 30-50 ml of water/100 g of DM was blended into the premixed ingredients. Then, with the help of a twin-screw meat grinder, the diets were prepared in the form of noodles with a 2 mm diameter. Upon air drying and sealing in Ziploc bags, the diets were preserved at -20°C in a refrigerator with <10% moisture content.

### 2.3. Fish and Breeding Management

Two thousand fish were brought from a nearby hatchery in Guangzhou, China. To prepare the fish for the trial, they were temporarily housed in a cement pond and fed commercial feed for three weeks prior to the trial. These fish were fasted for 24 h after the acclimatization period, and then a total of 720 healthy hybrid yellow catfish (17.15 ± 0.02 g; average ± standard deviation) were randomly distributed to 24 rectangular fiberglass tanks (length 98 cm × width 48.5 cm × height 38 cm) attached to a recirculating aquaculture system (RAS). The RAS system was equipped with a sedimentation basin, a biofilter, an ultraviolet sterilizer, temperature control, and continuous air circulation with air stones. Each experimental diet was then divided into three replicate aquaria of fish and served twice a day for 49 days at 08.15 and 16.15 hours until apparent satiation. Over the duration of the experiment, the water quality of the culture system was routinely checked, and the following values were acquired: temperature: 27.4 ± 0.5°C; dissolved oxygen: 6.0 ± 0.4 mg L^−1^; total ammonia nitrogen: 0.12 ± 0.02 mg L ^−1^, and pH: 7.1 ± 0.5. Keeping the photoperiod at 12 h : 12 h light : dark.

### 2.4. Production Performance and Condition Index

Following 49 days of feeding, fish were netted out of each tank, and the biomass of each tank was utilized to determine the production performance. Additionally, six fish per treatment were chosen at random, measured for body length, and dissected for liver and visceral weights to determine the condition factor (CF), intraperitoneal fat (IPF), hepatosomatic index (HSI), and viscerosomatic index (VSI). Both abdominal cavity fat and fat adhering to the intestinal tract of the fish were removed and weighed to ascertain the intraperitoneal fat. The parameters of growth performance and other morphology indices were calculated as per NRC [16].

### 2.5. Whole-Body Proximate Composition

At the beginning of the experiment, ten fish were taken as samples and stored at -20°C for a preliminary analysis of whole-body proximate composition. The whole fish was crushed in a meat grinder to create a homogeneous sample, which was then dried at 125°C for 48 hours. Five fish per treatment were sampled following the feeding trial and kept at 20°C until being tested. According to AOAC [46] guidelines, the moisture, crude protein, crude fat, and ash contents of a whole-body sample were estimated.

### 2.6. Plasma Antioxidants and Immune Analysis

Using 1 ml heparinized syringes, blood samples from six fish per treatment (two per tank) were taken (H6279; SIGMA) from the caudal vasculature for plasma analysis. After centrifugation at 3000 × g for 15 min at 4°C, plasma was separated and kept at -80°C for analysis. Plasma antioxidants and immune parameters (SOD, CAT, LMZ, and IgM) as well as glycogen were assayed by ELISA kits (Cusabio) as per the manufacturer's protocol.

### 2.7. Histological Examination of the Liver and Gut

Six fish per treatment (two per box) were taken for histological analysis of the liver and gut. Samples were fixed for 24 hours in 10% neutral-buffered formalin after being rinsed in a saline solution and subjected to histological examination. A series of dehydration processes in alcohol were followed for paraffin embedding. After that, a 5 mm section was taken from each block of embedded tissue. A standard staining procedure with hematoxylin-eosin was carried out on all gut sections, and oil red O staining was only performed on the liver. The average villus heights, enterocyte heights, muscular layer thicknesses, and serosa thicknesses per slice were determined using an image analysis system operated by a computer using a light microscope [47]. In each section, three measurements were taken for each parameter [48].

### 2.8. RNA Isolation and Reverse Transcription–Polymerase Chain Reaction (RT-PCR)

Six fish livers per treatment (two per box) were taken and kept at -80°C for expression of genes. Methods outlined in Wu et al. [47] were used to express mRNA in the liver. Briefly, to isolate liver RNA, TRIzol (Invitrogen) was used as directed by the manufacturer, and DNA contamination was removed from the RNA with RNA-Free DNase. Nanodrop spectrophotometry was used to determine RNA quantity and purity. In the next step, RNA was reversely transcribed to cDNA using a cDNA synthesis kit. Target of rapamycin (TOR) and insulin-like growth factor I (IGF-I) mRNA expressions in the liver were measured using quantitative real-time PCR (qPCR) using a StepOnePlus qPCR system from Applied Biosystems. The primers for the targeted genes and reference genes are listed in Table 2. The standard curve method was employed for the calculation of mRNA expression, and *β*-actin was used as a reference gene due to high stability across samples.

### 2.9. Statistical Analysis

A test of normal distribution and homogeneity of variance was performed on all data. The statistical significance of the tests was determined at a level of *P* < 0.05. The data were analyzed by one-way ANOVA and Tukey's multiple range test. Averages and standard deviations were used to express the data. Statistic data were evaluated using SPSS 18.00 (SPSS Inc.). The optimum dietary P/E ratio based on WG% was determined through the quadratic regression model. The oil red area was calculated under a light microscope (Olympus IX71) equipped with Image-Pro Plus 7.0 software.

## 3. Results

### 3.1. Growth Performance and Feed Utilization

**Figure 1:**
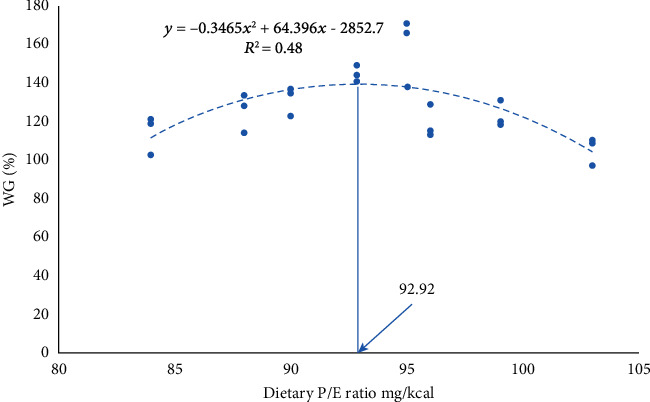
Effects of dietary P/E ratios on WG% of hybrid yellow catfish.

Table 3 shows the growth performance and feed utilization results of hybrid yellow catfish. At the end of the experiment, survival rates ranged from 98% to 100% with no significant changes across the groups (*P* > 0.05). The WG%, SGR, FCR, and DFI of hybrid yellow catfish were significantly (*P* < 0.05) affected by the dietary P/E ratio. With the amelioration in dietary P/E ratio from P/E84 to P/E95, significant (*P* < 0.05) enhancement in WG% and SGR was observed, while reduction in these parameters was noted by the further increase in dietary P/E ratio. The highest growth performance and feed utilization were observed in fish fed a P/E ratio of 95 mg/kcal. WG% of fish fed P/E95 was significantly higher compared to that of fish fed other dietary P/E ratios, while growth rates of fish fed P/E93 were comparable. Dietary P/E ratios significantly affected FCR and DFI (*P* < 0.05) with the lowest values recorded at the P/E95 level. When the dietary P/E ratio was increased up to 95 mg/kcal, the PER and PPV increased significantly (*P* < 0.05; Table 3). Based on the statistical analysis of WG% on the dietary P/E ratio, the optimal P/E ratio of hybrid yellow catfish was estimated to be 92.92 mg/kcal (Figure 1).

### 3.2. Morphometric Parameters

In the current study, hybrid yellow catfish fed varied P/E ratios did not produce any significant difference in their CF values (Table 4). Dietary P/E ratios significantly affected HSI (*P* < 0.05) with the lowest values recorded at P/E103. Similarly, the intraperitoneal fat (IPF) ratio increased as dietary P/E ratios decreased (*P* < 0.05).

### 3.3. Whole-Body Composition

Table 5 displays the whole-body proximate composition. In the current research work, total whole-body lipid content ameliorated in response to decreasing P/E ratios, whereas an inverse relationship was found for moisture content (*P* < 0.05). The whole-body protein and ash contents of the experimental fish were not affected by the ratio of dietary protein to energy (*P* > 0.05).

### 3.4. Antioxidant and Immunity-Related Parameters

The impact of varied dietary P/E ratios on both immune and antioxidative factors in hybrid yellow catfish is given in Table 6. The concentrations of lysozyme (LZM) and immunoglobulin (IgM) in the plasma of fish fed with P/E95 were significantly higher than those of fish fed with other dietary treatments (*P* < 0.05), and these two parameters were not significantly different when compared between fish fed P/E93 and fish fed P/E95 (*P* > 0.05). Fish fed with P/E95 displayed significantly higher concentration of superoxide dismutase (SOD) and catalase (CAT) in plasma than fish fed with other dietary P/E ratios (*P* < 0.05), and SOD or CAT concentration of fish fed with P/E93 were significantly lower than those of fish fed with P/E95.

### 3.5. Liver Histology and Glycogen Content

**Figure 2:**
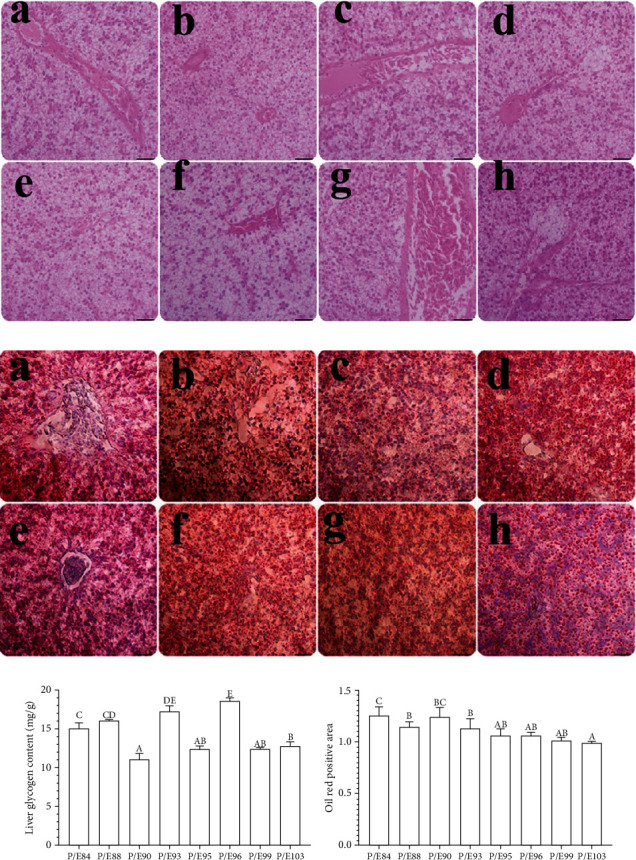
H&E staining (a). Oil red staining (b). Liver glycogen content (c). Oil red positive area (d). Liver histology among the different experimental groups. A P/E84, B P/E88, C P/E90, D P/E93, E P/E95, F P/E96, G P/E99, and H P/E103. Scale bar: 100 *μ*m.

The histological appearance of the liver is illustrated in Figure 2. It is observed that the dietary P/E ratios influence liver histology in hybrid fish (Figures 2(a)–2(d)). Lipid droplets were stained red with oil red staining. In comparison with the P/E 95, the P/E 84, P/E 88, and P/E 90 showed more intense lipid droplets (Figure 2(d)). Hepatic glycogen contents decreased with increasing gross energy, and higher protein diets showed comparatively lower values (*P* < 0.05; Figure 2(c)).

### 3.6. Gut Micromorphology

**Figure 3:**
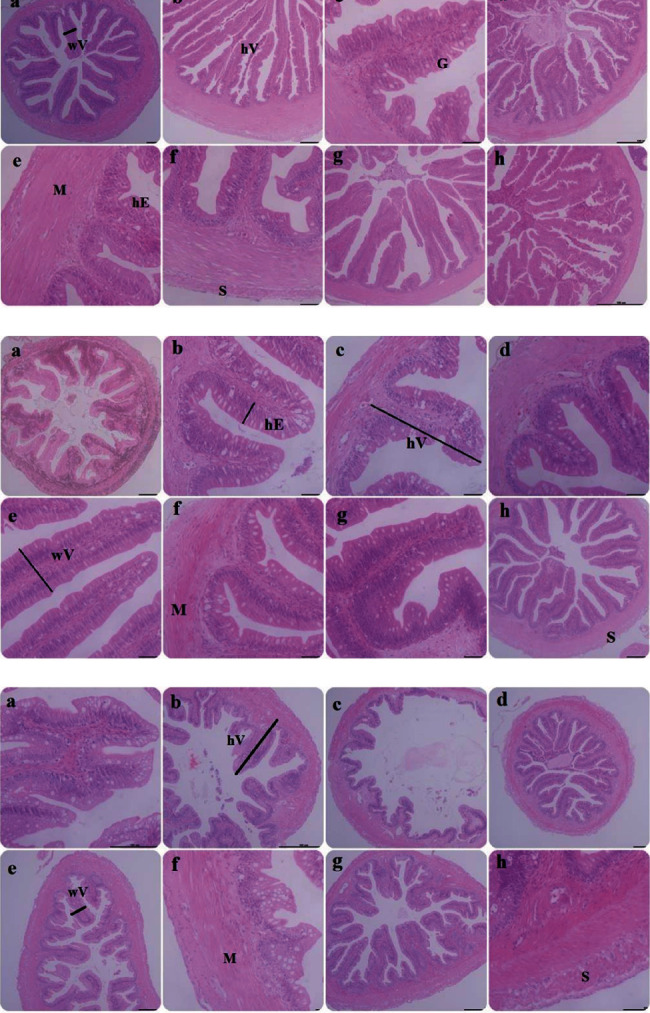
Histomorphology of the foregut (a), midgut (b), and Hindgut (c) of hybrid yellow catfish showing the histological structure in various dietary P/E ratio groups. The P/E ratio groups showed gradual enhancement of the intestinal villi. P/E ratios mg/kcal A P/E84, B P/E88, C P/E90, D P/E93, E P/E95, F P/E96, G P/E99, and H P/E103. hV: villus height; wV: villus width; hE: enterocyte height; M: muscular layer thickness; S: serosa thickness. Scale bar: 100 *μ*m.

A summary of the mircomorphometric measurements of the foregut, midgut, and hindgut is shown in Table 7 and Figure 3. The height of the villus, the height of enterocytes, the thickness of the muscular layer, and the thickness of the serosa of the gut all responded to different dietary P/E ratios (Figure 3; *P* < 0.05). Notably, fish treated with P/E95 had significantly larger villi in terms of both height and width (Table 7; *P* < 0.05).

### 3.7. Expression of Hepatic IGF-1 and TOR

**Figure 4:**
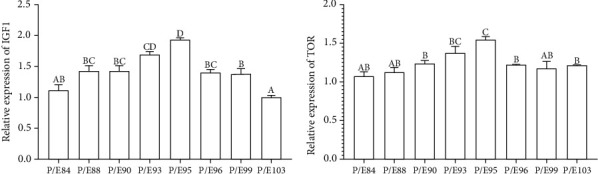
Effect of different dietary protein and energy ratios on relative expressions of IGF-1 (a) and TOR (b) genes in liver of hybrid yellow catfish. Values are means ± S.E.M. of three replications (*n* = 6). Values had different letters are significantly different (*P* < 0.05). IGF-1: insulin-like growth factor 1. TOR: target of rapamycin.

Figure 4 shows the expression of TOR and IGF-1 in the liver of hybrid yellow catfish. Dietary P/E ratios significantly upregulated the expression of IGF-1, with the P/E95 group having the highest value (*P* < 0.05). The gene expression of IGF-1 fed P/E93 was significantly lower than that of fish fed P/E95.

The relative expression of the TOR gene in the liver was significantly affected by different dietary P/E ratio groups (*P* < 0.05). The expression of TOR gene increased up to a certain point and thereafter declined as dietary P/E ratio increased, reaching the maximum at P/E 95.

## 4. Discussion

### 4.1. Optimum Dietary P/E Ratio Improved Growth

In this study, fish fed P/E 95 mg/kcal had optimum WG and SGR for fish. The fish fed P/E 93 had comparable WG and SGR to fish fed P/E95, but fish fed P/E95 had a lower FCR and significantly higher PER. In this case, it appears that 95 mg/kcal is an optimal dietary P/E ratio for hybrid yellow catfish. According to reports, the ideal dietary P/E ratio ranged from 98 to 145 mg/kcal for a number of different catfish species [49–52]. Therefore, the ideal P/E ratio in our study, 95 mg/kcal, is closely related to these fish species.

In the current research work, the weight gain of fish was also enhanced by the amelioration of protein levels. A maximum WG% was achieved for hybrid yellow catfish at 440 g/kg protein. Other catfish species consume similar amounts of protein, including Asian red-tailed catfish [53] and bagrid catfish [54]. The optimal dietary protein requirement for the yellow catfish has been reported to range between 37.58% and 39.02% [50] and 40.38% [41]. Moreover, a study on *Pelteobagrus vachelli* larvae has been conducted on lipid requirements [55]. While several existing studies have reported different protein requirements (320-440 g/kg of diet) of various catfish species [53, 54, 56–59], no information on the protein requirements or P/E ratios of hybrid yellow catfish is available to date. Hence, this study is a pioneering effort in this regard.

#### 4.1.1. Feed Utilization

In comparison to the low P/E ratio-fed fish, the high P/E ratio-fed fish had higher FCRs, which was parallel with the outcomes of prior investigations [51, 60, 61]. Fish fed with P/E95 achieved the most favorable FCR of 0.89 among the diets evaluated. This could be explained by the reason that the food conversion of the group fed the optimum P/E ratio was significantly better than that of the groups fed low and high P/E ratios. The fact that fish fed low-energy diets had considerably greater DFI than those fed other dietary treatments suggested that FI would increase on a low-energy diet. Fish adjust their feed intakes to match their energy needs [62]. Like most animals, fish regulate feed intake by the energy content of their diets [63, 64]. This process is typically seen as a way to obtain nutrients when dietary intake of an essential nutrient is inadequate. When given a high-energy diet, other fish species similarly showed a considerable decrease in feed intake [61, 65]. In this research, when the optimum P/E ratio (P/E95) was used, high PER/PPV were seen, indicating more growth per unit of protein taken. Comparable results have been reported for different fish species [8, 56]. Other investigations also revealed that both lipid and carbohydrate energy had a significant protein-sparing impact [58, 62, 66].

#### 4.1.2. Body Conditions Indices

The HSI provides information about the condition and nutritional status of fish. The present study showed that HSI decreased with increasing dietary P/E ratios, which might be due to the low nonprotein energy levels in the diets. In a study conducted by Gaylord and Gatlin III [67], they found that hybrid striped bass fed 20% lipids had a lower HSI than fish fed 5% or 10% lipids. In contrast, Lee et al. [68] report that rockfish fed 14% dietary lipid had a significantly higher HSI than those fed 7% dietary lipid. According to this study, high dietary lipid levels were associated with HSI. One notable exception was fish fed P/E96 which had a higher HSI despite being low in lipids. Also, fish fed P/E84 showed a notable increase in HSI compared to fish fed P/E90. This could be explained by the fact that in this trial, dietary starch ameliorates while dietary crude protein diminishes. HSI or hepatic glycogen always has a positive relation to digestible carbohydrate [67, 69]. In this study, hybrid yellow catfish fed high amounts of digestible carbohydrates had greater glycogen deposition in their liver. This is comparable to the existing literature on other fish species [70–73].

The IPF ratio increased as dietary P/E ratios decreased. The higher IPF ratio associated with higher dietary lipids indicates that excess lipid ingestion would lead to increased lipid accumulation in the body, which is in agreement with some other studies [74, 75]. In the same way, high VSI values can also be explained by high HSI and IPF values [76].

#### 4.1.3. Whole Body Composition

Inverse relationships existed between lipid content of carcass and dietary P/E ratio, and more dietary lipid or energy intake at each protein level led to higher carcass lipid content and lower carcass moisture levels, which was consistent with findings from other studies [41, 57]. In line with prior findings, ameliorated carcass lipid recommended that extra food energy from fat was partly stored as body fat [15, 21]. Furthermore, fish fed low-protein diets have ameliorated levels of lipids throughout their bodies. It appears that these outcomes are broadly in agreement with those of earlier studies on African catfish [49, 77]. On the other hand, the amount of protein or ash had no effect on the dietary P/E ratio. Ali and Jauncey [78] also reported similar outcomes for African catfish.

### 4.2. Optimum P/E Ratio Improved Immune and Antioxidative Ability

To find the best diets to support immunity and maintain the health status of hybrid yellow catfish, a variety of diets with varied dietary P/E ratios were designed in the current study. This study shows that dietary P/E ratios significantly affect the hybrid yellow catfish's nonspecific immune system. It has also been reported that *Pelteobagrus fulvidraco* displays similar results [55]. Fish innate immunity is regulated by biochemical molecules such as lysozyme (LZM) and immunoglobulins (IgM). The present findings revealed that fish fed with low or high P/E ratios showed a decrease in LZM and IgM concentrations in plasma, indicating that low or high P/E ratio diets can negatively affect immunity. Fish fed P/E 84 with higher lipid levels (170 g/kg) had lower plasma immunity levels. Similarly, channel catfish and European sea bass exhibited immunosuppression due to excessive dietary lipid intake [79, 80].

CAT and SOD are important components in the antioxidant defense system of fish [81, 82]. The oxidative status of fish is generally affected by a variety of factors, including nutritional factors [24]. There is, however, little data about how dietary energy affects oxidative status in fish [83]. Based on the variations in plasma concentrations of CAT and SOD in this study, it was found that the diet with P/E95 diminished the fish susceptibility to oxidation and might augment growth performance. In contrast, fish fed P/E93 show comparatively lower SOD and CAT concentrations than fish fed P/E95, which may be due to the elevated oxidation rates observed in carbohydrate-rich diets. Similar results were produced by Rueda-Jasso et al. [83] where lipid and carbohydrate energy sources influence the oxidative condition of *Senegalese sole*.

### 4.3. Optimum P/E Ratio Improved Liver and Intestinal Histomorphology

Fish histomorphology may offer an insight into how poor diets affect different tissues [84]. In the current research work, oil red staining showed that hybrid yellow catfish fed low P/E ratio diets had increased lipid buildup in their livers. Hepatocytes frequently produce plenty of large lipid droplets as a natural response to excess lipids [25]. The high levels of glycogen in fish fed P/E84, P/E88, P/E93, and P/E96 imply that glycogen storage may be responsible for hepatocyte vacuolization. Similar outcomes for *Labeo rohita* fry and *Catla catla* fingerlings were reported by Mohapatra et al. [85]. The overconsumption of carbohydrates could increase glycogen accumulation, increasing stress on the fish's metabolism.

It has been reported that the guts of vertebrates are highly reactive to modifications in the quality or quantity of nutrition provided by their diets [28]. A fish's ability to grow depends on the integrity of its gut because it serves as their main nutrient source [86, 87]. Intestinal tissue of fish receiving optimal P/E feedings displayed a distinctly developed histomorphology in the current investigation, with no obvious pathologic lesions. Fish have healthier guts and improved nutrition absorption capacity when their intestinal villi are longer. In turn, better digestive tracts result in improved fish performance [88]. The results show a considerable improvement in villus height and width in P/E95 based on gut morphometry. The histomorphometry analysis of a recent work by Kokou et al. [29] showed that the dietary protein-to-energy ratio affected intestinal health and ameliorated villus heights in juvenile shi drum. The ability of hybrid yellow catfish to digest their diets more effectively seems to be positively influenced by optimal dietary P/E ratios, improving their health, growth potential, and productivity.

### 4.4. Optimum Dietary P/E Ratio Increased Growth-Related Gene Expression

Growth hormone, which is regulated by a mitogenic peptide-like insulin growth factor 1 or IGF-I, plays a major role in controlling fish growth [89, 90]. A number of animal organs (the gut, liver, or kidney) synthesize IGF-I. But in fish, liver IGF-I is thought to be one of the most important growth mediators [91, 92]. The nutritional condition of numerous fish species has a significant impact on hepatic IGF-1 levels [91, 93]. In current research, hepatic IGF-I expression was directly correlated with the optimum P/E ratio; however, as the P/E ratio increases or decreases further, the expression level of IGF-I diminishes. Increasing protein intake had a similar effect on IGF-I expression as an increased dietary P/E ratio did, supporting the significance of IGF-I regulation in fish WG% or growth as documented by Qiang et al. [94] and Singha et al. [92] in Nile tilapia. Studies on a variety of fish, including GIFT [92] and salmonids [95], demonstrated a substantial link between growth hormone IGF-I and growth. Related findings indicated that the IGF-I expression followed a pattern that was similar to WG% [91]. It was found that the combined effects of crude protein, fat, and carbohydrate considerably influenced IGF-I. Besides, Moriyama et al. [96] previously established the function of IGF-I in the metabolism of fish proteins, lipids, carbohydrates, and minerals.

TOR is a conservation-rich protein kinase that is essential for inhibiting translation and increasing peptide or protein formation [97]. In the present research, P/E93 had notably lower levels of TOR mRNA than P/E95. This outcome might be the result of a lack of dietary protein limiting protein formation by blocking the gene TOR signaling. The result falls in line with earlier research on Chinese perche consuming low-protein diets, which revealed the expression of mTOR was suppressed specifically in the liver [98]. The findings of the combined culture also revealed that the low protein diets had reduced total fish body protein content and PPV, as well as TOR signalling pathway suppression.

## 5. Conclusions

These results suggest that a diet having a P/E ratio of 95 mg per kcal (440 g of protein per kg of dry matter and 150 g of lipid per kg of dry matter along with 4.56 kcal per g of GE) would be appropriate to maximize growth in hybrid yellow catfish. Moreover, low or high P/E ratios showed a negative impact on nonspecific immunity, oxidative stress, and liver function. Additionally, the expression of the TOR and IGF-1 genes in the liver was upregulated by an optimal dietary protein-to-energy ratio. Collectively, the outcomes of the study clearly demonstrated that a 92.92 mg/kcal dietary P/E ratio could improve growth performance, immune response, antioxidant capacity, and promote growth through the TOR and IGF1 genes.

## Figures and Tables

**Table 1:** Formulations and analyzed composition of experimental diets.

P/E ratios	Dietary treatments							
	84	88	90	93	95	96	99	103
Fish meal	220	220	220	220	220	220	220	220
Poultry by-product meal	70	70	110	70	110	70	110	110
Soya bean meal	195	195	230	195	230	195	230	230
Cottonseed protein meal	40	40	70	40	70	40	70	70
Cassava starch	0	0	40	0	40	0	40	40
Wheat flour	256.5	256.5	160	256.5	160	256.5	160	160
Soybean oil	110	90	110	70	90	50	70	50
Lecithin oil	10	10	10	10	10	10	10	10
Monocalcium phosphate	20	20	20	20	20	20	20	20
Deglued bone meal	51	71	2.5	91	22.5	111	42.5	62.5
Mildew preventive	1.3	1.3	1.3	1.3	1.3	1.3	1.3	1.3
Additive premix (V+M)	10	10	10	10	10	10	10	10
L-Lys hydrochloride	5	5	5	5	5	5	5	5
DL-met	2.5	2.5	2.5	2.5	2.5	2.5	2.5	2.5
L-threonine	2.5	2.5	2.5	2.5	2.5	2.5	2.5	2.5
Choline chloride (50%)	3	3	3	3	3	3	3	3
Lutein	3	3	3	3	3	3	3	3
Antioxidant	0.2	0.2	0.2	0.2	0.2	0.2	0.2	0.2
Analyzed composition of diets								
Dry matter	928.2	917.2	912.1	909.1	909.7	908.0	908.1	909.1
Ash	19.9	21.8	16.2	24.2	18.7	26.7	16.7	14.5
Crude protein	392.4	397.3	430.8	399.7	435.2	397.5	437.2	439.1
Crude lipid	168.5	150.3	165.7	128.7	148.5	111.7	128.1	114.6
Total starch	204.7	205.1	170.7	198.4	166.0	204.5	166.8	174
Gross energy kcal/g	4.66	4.48	4.76	4.29	4.56	4.13	4.40	4.26
P/E mg/kcal	84.20	88.68	90.50	93.17	95.43	96.24	99.36	103.07

Fish meal: 64.99% CP, 4604.64 kcal/kg CE, and 9% CL. Poultry by-product meal: 63.57% CP, 4814.97 kcal/kg CE, and 10.77% CL. Soya bean meal: 37.69% CP, 4324.06 kcal/kg CE, and 2.04% CL. Cottonseed protein meal: 60.56% CP, 4554.14 kcal/kg CE, and 1.62% CL. Cassava starch: 0.17% CS, 3780.52 kcal/kg CE, 0.3% CL, and 89.06% CS. Wheat flour: 17.50% CS, 4244.08 kcal/kg CE, 2.69% CL, 60.55% CS. Soybean oil: 9261.00 kcal/kg CE and 98% CL. Lecithin oil: 6860.70 kcal/kg CE and 72.6% CL. Deglued bone meal: 9.51% CP, 1069.49 kcal/kg CE, and 5.24% CL. L-lys hydrochloride: 86.4% CP, 5235.06 kcal/kg CE, and 78% Lys. DL-Met: 57.2% CP, 4785.38 kcal/kg CE, and 99% Met; L-threonine: 73.1% CP, 4130.15 kcal/kg CE, and 99% Thr, supplied by the ChengYi Co. Ltd., Guangzhou, China. Vitamin and mineral premix (mg-1 kg of product): vitamin A, 3000000 IU; vitamin D3,15000IU; vitamin E, 4000; vitamin K3, 450; vitamin B1, 800; vitamin B2, 850; vitamin B6, 600; vitamin B12, 1.5; folic acid, 130; inosi, 4000; L-ascorbate-2-phosphate, 11000; nicotinamide, 3000; calcium pantothenate, 1700; D-biotin, 15; D-calcium pantothenate, 1700; Cu, 650; Fe, 4500; Mn, 850; Zn, 5300; I, 120; Se, 35; Co, 100, supplied by the ChengYi Co. Ltd., Guangzhou, China.

**Table 2:** Primers used for determining gene expression of hybrid yellow catfish (qPCR).

Used for	Gene name	Gene bank accession no.	Primer sequence (5′-3′)
qPCR	IGF1^1^	XM_027160377.2	^3^F: CATCAGCCAAGTCTGGTGGT
			^4^R: CAGATGTTCCCTCACCATCCT
	TOR^2^	XM_027166728.2	F: GAAGGACCTGACTCAAGCCT
			R: ATTGGCTGGTTGGGGTCATA
	*β*-Actin	XM_027148463.1	F: GAAGGTTATGCTCTGCCCCAT
			R: GTGAAGCTGTAGCCTCTCTCG

^1^IGF 1: insulin-like growth factor-1; ^2^TOR: target of rapamycin; ^3^F: forward sequence; ^4^R: reverse sequence.

**Table 3:** Growth performance and feed utilization of hybrid yellow catfish fed different dietary P/E ratios for 7 weeks.

P/E ratio mg/kcal	IBW (g)^1^	FBW (g)^2^	WG (%)^3^	SGR^4^	FCR^5^	DFI^6^	PER^7^	PPV^8^	Survival %
84	17.0 ± 0.0	37 ± 2^a^	114 ± 9^a^	1.55 ± 0.10^ab^	1.09 ± 0.05^bc^	1.75 ± 0.55^ab^	2.51 ± 0.12^bc^	38 ± 2^ab^	100
88	17.1 ± 0.1	39 ± 2^ab^	125 ± 10^ab^	1.65 ± 0.10^abc^	1.07 ± 0.05^bc^	1.81 ± 0.61^abc^	2.58 ± 0.11^bcd^	43 ± 4^bc^	100
90	17.1 ± 0.1	40 ± 1^abc^	131 ± 7^abc^	1.72 ± 0.07^bcd^	1.05 ± 0.01^bc^	1.85 ± 0.60^abc^	2.43 ± 0.02^bc^	37 ± 1^ab^	100
93	17.0 ± 0.0	42 ± 1^bc^	144 ± 4^bc^	1.83 ± 0.03^cd^	1.02 ± 0.03^ab^	1.89 ± 0.53^bc^	2.69 ± 0.07^cd^	50 ± 3^cd^	98
95	17.0 ± 0.0	43 ± 3^c^	158 ± 17^c^	1.93 ± 0.14^d^	0.89 ± 0.07^a^	1.74 ± 0.08^a^	2.84 ± 0.21^d^	55 ± 3^d^	100
96	17.1 ± 0.0	38 ± 2^ab^	119 ± 9^ab^	1.60 ± 0.08^abc^	1.18 ± 0.09^cd^	1.94 ± 0.54^c^	2.36 ± 0.18^bc^	39 ± 4^b^	100
99	17.2 ± 0.1	38 ± 1^ab^	123 ± 7^ab^	1.64 ± 0.06^abc^	1.10 ± 0.05^bc^	1.86 ± 0.22^abc^	2.29 ± 0.10^ab^	39 ± 1^b^	100
103	17.2 ± 0.1	35 ± 1^a^	105 ± 7^a^	1.47 ± 0.07^a^	1.27 ± 0.06^d^	1.92 ± 0.24^c^	1.97 ± 0.09^a^	30 ± 2^a^	100

Values are means ± S.E.M. of three replications. Values in the same line with superscripts are significantly different (*P* < 0.05). IBW^1^: initial body weight; FBW^2^: final body weight; WG (%)^3^:weight gain; SGR^4^: specific growth rate, % day^−1^; FCR^5^: feed conversion ratio; DFI^6^:daily feed intake; PER^7^: protein efficiency ratio; PPV^8^: protein productive value.

**Table 4:** Body condition indices of hybrid yellow catfish fed different dietary P/E ratios for 7 weeks.

P/E ratio mg/kcal	CF^1^	VSI^2^	HSI^3^	IPF^4^
84	1.87 ± 0.02	6.99 ± 0.39^c^	1.41 ± 0.04^b^	2.99 ± 0.36^c^
88	1.90 ± 0.04	7.03 ± 0.28^c^	1.43 ± 0.05^b^	3.06 ± 0.43^c^
90	1.87 ± 0.03	6.50 ± 0.56^bc^	1.38 ± 0.11^ab^	2.54 ± 0.47^bc^
93	1.86 ± 0.06	5.78 ± 0.37^ab^	1.31 ± 0.06^ab^	1.98 ± 0.39^ab^
95	1.98 ± 0.14	6.47 ± 0.28^bc^	1.33 ± 0.09^ab^	2.45 ± 0.05^bc^
96	1.94 ± 0.05	5.82 ± 0.63^ab^	1.49 ± 0.11^b^	1.96 ± 0.36^ab^
99	1.86 ± 0.06	5.78 ± 0.37^ab^	1.31 ± 0.06^ab^	1.98 ± 0.39^ab^
103	1.84 ± 0.09	5.15 ± 0.35^a^	1.16 ± 0.09^a^	1.16 ± 0.26^a^

Values are means ± S.E.M. of three replications. Values in the same line with superscripts are significantly different (*P* < 0.05). CF^1^: condition factor; VSI^2^: viscerosomatic index; HSI^3^: hepatosomatic index; IPF^4^: intraperitoneal fat ratio.

**Table 5:** Whole Body composition (g/kg, wet basis) of hybrid yellow catfish fed different dietary P/E ratios for 7 weeks.

P/E ratio mg/kcal	Moisture	Lipid	Protein	Ash
84	724.1 ± 4.4^a^	80.3 ± 7.7^c^	140 ± 7	35.9 ± 2.1
88	728.7 ± 2.7^ab^	79.8 ± 6.5^c^	150 ± 6	31.2 ± 2.0
90	733.4 ± 9.4^abc^	74.9 ± 9.1^bc^	150 ± 1	39.1 ± 0.7
93	733.6 ± 4.4^abc^	73.4 ± 7.9^bc^	159 ± 6	30.3 ± 4.6
95	734.3 ± 0.8^abc^	71.0 ± 5.7^bc^	160 ± 5	34.2 ± 4.2
96	744.1 ± 2.3^bc^	65.6 ± 3.8^ab^	150 ± 8	35.7 ± 0.1
99	744.2 ± 2.5^bc^	63.8 ± 5.1^ab^	150 ± 4	33.3 ± 0.4
103	749.2 ± 3.1^c^	53.7 ± 4.8^a^	140 ± 7	36.6 ± 1.9

Values are means ± S.E.M. of three replications. Values in the same line with superscripts are significantly different (*P* < 0.05). Initial whole body composition (g/kg): moisture = 744.8; protein = 135.8; lipid = 42.8; Ash = 37.6.

**Table 6:** Plasma oxidative & immunological status of hybrid yellow catfish fed different dietary P/E ratios for 7 weeks.

P/E ratio mg/kcal	LZM^1^ (ng/ml)	IgM^2^ (ug/ml)	SOD^3^ (ng/ml)	CAT^4^ (U/ml)
84	2470 ± 337^a^	60 ± 8^a^	1304 ± 23^ab^	472 ± 26^b^
88	3117 ± 308^ab^	66 ± 3^ab^	1303 ± 99^ab^	451 ± 17^b^
90	3146 ± 151^ab^	64 ± 8^ab^	1640 ± 84^cd^	497 ± 30^bc^
93	4011 ± 178^b^	74 ± 5^b^	1478 ± 94^bc^	475 ± 32^b^
95	4216 ± 150^b^	79 ± 7^b^	1699 ± 91^d^	565 ± 50^c^
96	3192 ± 167^ab^	66 ± 9^ab^	1118 ± 72^a^	343 ± 10^a^
99	3926 ± 168^b^	71±6^ab^	1329 ± 31^b^	457 ± 17^b^
103	3222 ± 574^ab^	72 ± 3^ab^	1108 ± 74^a^	336 ± 29^a^

Values are means ± S.E.M. of three replications. Values in the same line with superscripts are significantly different (*P* < 0.05). LZM^1^: lysozyme; IgM^2^: immunoglobulin; SOD^3^: superoxide dismutase; CAT^4^: catalase.

**Table 7:** The gut micromorphology of hybrid yellow catfish fed different dietary P/E ratio for 7 weeks.

P/E ratio mg/kcal	Foregut (*μ*m)					Midgut (*μ*m)					Hindgut (*μ*m)				
	hV^1^	wV^2^	hE^3^	M^4^	S^5^	hV	wV	hE	M	S	hV	wV	hE	M	S
84	202 ± 5^ab^	27 ± 3^a^	16 ± 1^c^	32 ± 2^a^	13 ± 2^ab^	107 ± 10^b^	25 ± 4^ab^	7 ± 1^ab^	14 ± 2^ab^	12 ± 2^ab^	81 ± 8^a^	26 ± 2^ab^	7 ± 1^ab^	16 ± 1^a^	13 ± 1^ab^
88	216 ± 6^ab^	28 ± 4^ab^	16 ± 1^c^	34 ± 4^ab^	13 ± 3^ab^	115 ± 4^bc^	27 ± 3^abc^	7 ± 1^ab^	13 ± 3^a^	12 ± 2^ab^	104 ± 5^bc^	30 ± 3^ab^	7 ± 1^abc^	17 ± 1^ab^	12 ± 1^ab^
90	219 ± 12^b^	37 ± 3^bc^	16 ± 0^c^	31 ± 6^a^	15 ± 2^b^	117 ± 6^bc^	23 ± 2^a^	6 ± 2^a^	19 ± 3^b^	12 ± 1^ab^	103 ± 5^bc^	34 ± 3^bc^	6 ± 1^a^	18 ± 1^ab^	13 ± 1^ab^
93	302 ± 15^c^	39 ± 7^c^	20 ± 1^d^	41 ± 3^bc^	12 ± 2^ab^	128 ± 6^cd^	31 ± 4^bc^	8 ± 1^ab^	18 ± 2^bc^	16 ± 3^bc^	120 ± 10^cd^	36 ± 3^bc^	9 ± 1^cd^	21 ± 3^bc^	16 ± 1^c^
95	328 ± 16^d^	40 ± 3^c^	21 ± 2^d^	45 ± 2^c^	16 ± 2^b^	134 ± 11^d^	34 ± 1^d^	9 ± 2^b^	19 ± 2^b^	17 ± 2^c^	128 ± 10^d^	38 ± 2^c^	10 ± 1^d^	23 ± 2^c^	17 ± 1^c^
96	214 ± 12^ab^	27 ± 3^a^	12 ± 1^ab^	32 ± 2^a^	13 ± 2^ab^	118 ± 7^bc^	25 ± 2^ab^	7 ± 1^ab^	13 ± 2^a^	11 ± 2^ab^	104 ± 6^bc^	30 ± 1^ab^	8 ± 1^bcd^	17 ± 1^ab^	13 ± 1^ab^
99	218 ± 15^b^	28 ± 5^ab^	12 ± 1^a^	33 ± 5^a^	12 ± 2^ab^	110 ± 3^b^	29 ± 3^abc^	7 ± 1^ab^	13 ± 1^a^	10 ± 2^a^	101 ± 11^b^	30 ± 2^ab^	6 ± 1^a^	18 ± 1^abc^	13 ± 1^b^
103	199 ± 11^a^	26 ± 3^a^	15 ± 2^bc^	32 ± 4^a^	10 ± 1^a^	91 ± 5^a^	26 ± 2^ab^	5 ± 0^a^	12 ± 1^a^	9 ± 2^a^	82 ± 11^a^	27 ± 5^a^	7 ± 1^abc^	14 ± 2^a^	11 ± 1^a^

Values are means ± S.E.M. of three replications. Values in the same line with superscripts are significantly different. hV^1^: villus height; wV^2^: villus width; hE^3^: enterocytes height; M^4^: muscular layer thickness; S^5^:serosa thickness.

## Data Availability

Upon reasonable request, the corresponding author will provide supporting data.
